# Fortification of Goat Milk Yogurts with Encapsulated Postbiotic Active Lactococci

**DOI:** 10.3390/life14091147

**Published:** 2024-09-11

**Authors:** Andrea Lauková, Marián Maďar, Natália Zábolyová, Aleksandra Troscianczyk, Monika Pogány Simonová

**Affiliations:** 1 Centre of Biosciences of the Slovak Academy of Sciences, Institute of Animal Physiology, Šoltésovej 4-6, 040 01 Košice, Slovakia; kotesovska@saske.sk (N.Z.); simonova@saske.sk (M.P.S.); 2 Department of Microbiology and Immunology, University of Veterinary Medicine and Pharmacy in Košice, Komenského 73, 041 84 Košice, Slovakia; marian.madar@uvlf.sk; 3 Sub-Department of Veterinary Microbiology, Faculty of Veterinary Medicine, University of Life Sciences in Lublin, Akademicka Street 12, 20 950 Lublin, Poland; aleksandra.troscianczyk@up.lublin.pl

**Keywords:** goat milk, bacteriocin, dairy products, supplementation, lactococci

## Abstract

The species *Lactococcus lactis* is a bacterium extensively used in the dairy industry. This bacterium is Generally Recognized as Safe and was added to the European Food Safety Authority’s Qualified Presumption of Safety list. The major functions of this species in dairy fermentation are the production of lactic acid from lactose, citric acid fermentation, and the hydrolysis of casein. But, the representatives of this species that produce bacteriocin substances can also exert an inhibitory effect against spoilage bacteria. The aims of this study were to test three lactococcal strains isolated from raw goat milk for their postbiotic activity and to test their stability in goat milk yogurts after their application in encapsulated form for their further application. To achieve these aims, validated methods were used. Three *Lactococcus lactis* strains (identified by Blastn 16S rRNA analysis) produced bacteriocin substances/postbiotics. These concentrated postbiotics inhibited the growth of enterococci and staphylococci (by up to 97.8%), reaching an inhibitory activity of up to 800 AU/mL. The encapsulated (freeze-dried) lactococci survived in the goat milk yogurts with sufficient stability. Strain MK2/8 fortified the yogurts in the highest amount (8.1 ± 0.0 cfu/g log 10). It did not influence the pH of the yogurt.

## 1. Introduction

Lactococci represent one of the most important groups of lactic acid bacteria (LAB) used in the dairy industry, especially the species *Lactococcus lactis* [1,2]. The species *L. lactis* belongs to the phylum Bacillota (synonym: Firmicutes), the class Bacilli, the order Lactobacillales, the family Streptococcacae, and the genus *Lactococcus* [3]. The synonym Firmicutes for Bacillota (lactococci), introduced in 2021, remains controversial among microbiologists, so both names can be used [4]. Nowadays, twelve species of *Lactococcus* are recognized: *Lactococcus allomyrinae*, *L. carnosus*, *L. chungangensis*, *L. cremoris* with the two subspecies *cremoris* and *tructae*, *L. formosensis* with the subspecies *bovis* and *formosensis*, *L. fujiensis*, *L. garviae* with the subspecies *garviae* and *bovis*, *L. hircilactis*, *L. hodotermopsidis*, *L. insecticola*, *L. kimchi*, *L. lactis* with the subspecies *lactis* and *hordniae*, *L. laudensis*, *L. nasutitermitis*, *L. paracarnosus*, *L. petauri*, *L. piscium*, *L. plantarum*, *L. protaetiae*, *L. raffinolactis*, *L. reticulitermitis*, *L. taiwanensis*, and *L. termiticola* [5]. Special focus is placed on the representatives of the species *L. lactis*, which are used in the processing of fermented dairy products, such as yogurts, cheeses, and/or cream. Fermented foods have been understood to be healthy foods that play an important role in the maintenance of human health [6]. Some representatives of the species *L. lactis* have been demonstrated to be promising candidates for the delivery of functional proteins because of their non-invasive and non-pathogenic characteristics [7]. Lactococci have a long documented history of safe use, supported by their designation as Generally Recognized as Safe by the Food and Drug Administration and Qualified Presumption of Safe (QPS) by the European Food Safety Authority (EFSA) [8]. Moreover, the species *L. lactis* is one of the best-characterized low-GC Gram-positive bacteria, with detailed knowledge of its genetics, metabolism, and biodiversity [9,10]. Besides the suitable technological functions of some representatives of the species *L. lactis*, there are also strains with probiotic and/or postbiotic characteristics (bacteriocin-producing strains). As is well known, probiotics are defined as non-pathogenic bacterial strains that induce health benefits in the host when ingested in adequate amounts [11]. These beneficial strains can possess postbiotic (bacteriocin) activity. This means that they can produce bacteriocins (substances of proteinaceous character) with antimicrobial (inhibitory) activity [12]. The bacteriocin-producing, beneficial/probiotic strains have an advantage in competitive interactions with spoilage bacteria in food matrices [13].

Recently, bacteriocins have been added to the group of postbiotics. Postbiotics are defined as preparations of inanimate microorganisms and/or their components that confer a health benefit to the host [14,15]. Products made from goat milk are popular among the population in Slovakia and are frequently consumed, and goat husbandry, as well as goat milk production and processing, has a long tradition in Slovakia. The implementation of postbiotic, active, beneficial strains in diverse food products contributes substantially to food protection, food safety, and food security. Moreover, these strains or their bacteriocins often support, e.g., the immune system [16,17]. Therefore, yogurts fortified with the mentioned bacteriocin-producing (postbiotic active) strains are considered functional foods that have health-promoting and -supporting benefits for consumers. Some examples of functional foods are iodized salt, vitamin-A- and -D-fortified milk and yogurt, and folic-acid-enriched bread. However, for each, the application form of the additive is important because a simple, economically available and protective method is the most requested. It seems that encapsulation meets these requirements. Encapsulation is a process/tool used to improve the delivery of bioactive molecules and living cells to foods [18]. One of the most important reasons for the encapsulation of active ingredients is to provide improved stability in the final products and during processing. Freeze-drying is the simplest form of encapsulation [19,20]. The aims of this study were to test the postbiotic activity of lactococci isolated from raw goat milk and their stability in goat milk yogurts after their application in encapsulated form.

## 2. Materials and Methods

### 2.1. Isolation and Identification of Lactococci

Twenty-seven raw goat milk samples were collected from healthy goats in the central region of Slovakia, as previously described by Lauková et al. [21]. To treat the milk samples, the standard microbiological dilution method was applied, as specified by the International Organization for Standardization (ISO). Raw goat milk samples were diluted in Ringer solution (pH 7.0, Merck, Darmstadt, Germany). Dilutions were plated onto MRS agar (De Man–Rogosa–Sharpe agar, Merck, Darmstadt, Germany, pH 6.3), BHI agar (pH 7.0, Difco-Becton Dickinson company, Sparks, MD, USA), and/or M17 agar (Difco-Becton Dickinson, Sparks, MD, USA, pH 6.9) and incubated at 37 °C for 24/48 h to detect lactococcal colonies. Then, picked-up colonies were checked for purity using BHI agar (Difco-Becton Dickinson, Sparks, MD, USA) enriched with sheep blood, and, based on their technological properties [22], three strains were selected for more detailed study: MK2/2, MK2/7, and MK2/8 (in co-operation with our colleagues from the Dairy Research Institute in Žilina, Slovakia). In our laboratory, these strains were checked for hemolysis using the method described by Lauková et al. [21] and Semedo-Lemsaddek et al. [23]. As the next step, the strains were submitted for sequence analysis.

### 2.2. DNA Extraction, PCR Amplification, and Sequencing

A detailed description of the method was provided in our previous study [24]. Briefly, the genomic DNA was extracted from pure colonies by using DNAzol direct (Molecular Research Centre Inc., Cincinnati, OH, USA) following the instructions of the manufacturer. The 16S ribosomal RNA genes from isolates were amplified by PCR using universal primers (Merck-Sigma Aldrich, Darmstadt, Germany) such as Bac27F (5-AGAGTTTGATCMTGGCTCAG-3) and 1492R (5-CGGYTACCTTGTTACGACTT-3). The PCR mixture (50 μL) contained 2 μL of DNA shield and 46 μL of a reaction mixture comprising One Taq 2× Master Mix with Standard Buffer (New England Biolabs, Hitchin, UK), diluted in water for molecular biology (PanReac AppliChem, Darmstadt, Germany) to a 1× concentration, and 1 μL of each primer (concentration 33 μM). The PCR conditions (thermocycler—TProfesional Basic, Biometra GmbH, Göttingen, Germany) used were as follows: 94 °C for 5 min, followed by 30 cycles of denaturation at 94 °C for 1 min, annealing at 55 °C for 1 min, and primer extension at 72 °C for 3 min, and then a final step at 72 °C for 10 min. Aliquoted PCR products were separated by horizontal 3% (*w*/*v*) agarose gel electrophoresis in Tris-acetate-EDTA buffer (pH 7.8) and visualized with GelRed (Biotium, Inc., Hayward, CA, USA). Amplified products in low-binding tubes with a minimum volume of 15 µL were sent for purification and sequencing in both directions using 1492R and Bac27F primers (Microsynth, Wien, Austria). The 16S rRNA sequence was validated and assembled by Geneious 8.0.5 (Biomatters, Auckland, New Zealand) and subjected to BLASTn analysis (https://doi.org/10.1093/nar/gkh435, accessed on 1 July 2004).

### 2.3. Enzymatic Profiles of Selected Lactococci

The following enzymes involved in the API-ZYM panel system (BioMerieux, Marcy lEtoile, France) were tested: alkaline phosphatase, esterase (C4), esterase lipase (C8), lipase (C14), leucine arylamidase, valine arylamidase, cystine arylamidase, trypsin, α-chymotrypsin, acid phosphatase, naphtol-AS-BI-phosphohydrolase, α-galactosidase, β-galactosidase, β-glucuronidase, α-glucosidase, β-glucosidase, N-acetyl-β-glucosaminidase, α-mannosidase, and α-fucosidase. The method was previously described by Lauková et al. [21]. Briefly, a volume of 65 µL of the tested strain inoculum (MacFarland stage 1) was transferred into each well of the test panel plate. The panel plate was incubated at 37 °C for 4 h. Then, one drop each of Zym A and Zym B reagents was added to each well. Enzyme activity was assessed according to a color intensity evaluation (0–5). A relevant value in nanomol (nmoL) was assigned to each reaction according to the color chart supplied with the kit.

### 2.4. Susceptibility to Antimicrobials

Susceptibility to antimicrobials (antibiotics) was assayed using two methods: the agar diffusion test with antibiotic disks and E-test/strip diffusion, as recommended by EUCAST (European Committee on Antimicrobial Susceptibility Testing System) [25].

Using the agar diffusion method, BHI agar enriched with blood (Difco-Becton Dickinson, Sparks, MD, USA) was used. Broth cultures of testing strains (100 µL) were spread on the agar surface, and the following antibiotic disks (12) were tested (as recommended by disk supplier): clindamycin (2 µg), novobiocin (5 µg), penicillin (10 IU), ampicillin (10 µg), erythromycin (15 µg), streptomycin (25 µg), rifampicin (30 µg), vancomycin (30 µg), kanamycin (30 µg), chloramphenicol (30 µg), ticarcillin (75 µg), and gentamicin (120 µg). Disks were supplied by Oxoid Limited (Basingstoke, Hampshire, United Kingdom), except for kanamycin, which was supplied by Lachema company (Brno, Czech Republic). The agar plates with disks were cultivated overnight at 37 °C, and then susceptibility (inhibitory zone diameter) or resistance to antibiotics was evaluated according to EUCAST.

In the case of the E-strip method, the minimum inhibitory concentration (MIC) was established using the following antibiotic strips: penicillin (0.016–256 µg/mL), chloramphenicol (0.016–256 µg/mL), gentamicin (0.064–1024 µg/mL), rifampicin (0.032–32 µg/mL), streptomycin (0.064–1024 µg/mL), erythromycin (0.015–256 µg/mL), kanamycin (0.016–256 µg/mL), vancomycin (0.016–256 µg/mL), tetracycline (0.016–256 µg/mL), and ampicillin (0.016–256 µg/mL). M17 agar (Difco) plates were seeded with overnight broth cultures (BHI, Difco) of the tested strains (100 µL). Antibiotic strips were placed on the surfaces of the plates. *E. faecalis* ATCC2921 and/or *Lactococcus lactis* CCM 1881 were included as positive control strains.

### 2.5. Biofilm-Forming Ability of Lactococci

This parameter was tested using a quantitative plate assay according to Chaieb et al. [26] and Slížová et al. [27], as previously described by Lauková et al. [21]. A pure colony of the strain grown on M17 agar (Difco) overnight at 37 °C was inoculated into 5 mL of Ringer solution (pH 7.0, 0.75% *w*/*v*). The suspension corresponded to 1 × 10^8^ cfu/mL. A volume of 100 µL from this dilution was transferred into microtiter plate wells (Greiner ELISA 12 well strips, Frickenhausen GmbH, Frickhausen, Germany). After the incubation of the plate for 24 h at 37 °C, the biofilm forming in the microtiter plate well was washed twice with 200 µL of deionized water and dried at room temperature for 40 min. The staining of attached bacteria was performed with 200 µL of 0.1% crystal violet in deionized water at 25 °C for half an hour. Then, the dye solution was aspirated away, and the wells were washed twice with 200 µL of deionized water. After water removal, the plate was dried for half an hour at room temperature. The dye bound to the adhered biofilm was extracted using 200 µL of 95% ethanol. A volume of 150 µL was then transferred from each well to a new microtiter plate for absorbance (A_570_) measurement with the use of the Apollo 11 LB 913 absorbance reader (Apollo, Berthold-technologies, Oak Ridge, TN, USA). Two independent runs with 12 replicates were measured, also including a negative control (BHI broth). *Streptococcus equi* subsp. *zooepidemicus* CCM 7316 was the positive control (provided by Dr. Styková from the University of Veterinary Medicine and Pharmacy in Košice, Slovakia). The biofilm-forming ability of lactococci was evaluated according to the following classification criteria [22,26,27]: highly positive (A570 ≥ 1.0), low-grade positive (0.1 ≤ A570 ≤ 1.0), and negative (A570 ≤ 0.1).

### 2.6. Concentrated Bacteriocin Substance Preparation and Postbiotic Activity Testing

Lactococci (0.1% inoculum) were inoculated in a volume of 40 mL of MRS broth (Merck, Darmstadt, Germany, pH 6.5–7). They were incubated at 37 °C for 24 h to reach an A_600_ of up to 1.0 (MK2/2-0.834, MK2/7-0.977, MK2/8-0.984). The grown cultures were centrifuged (10,000× *g*) for half an hour. The pH of the supernatants was adjusted to 5.5–6.0. The cell-free supernatants were treated by adding EDTA/Chelaton III (Sigma-Aldrich, Muenchen, Germany) and heated at 80 °C for 10 min to eliminate the effects of other organic substances. Then, they were concentrated using Concentrator Plus (Eppendorf, Hamburg, Germany) to obtain concentrated substances (4.0 mL, CBs). The inhibitory activity was tested using an agar spot test [28]. Briefly, as the bottom agar layer, BHI agar (Difco) was used, and as semi-solid agar for the surface overlay, 0.7% M17 agar was used (Difco). The inhibitory activity was expressed in arbitrary units per mL (AU/mL) and corresponds to the highest dilution of concentrated substances that caused the growth inhibition of the indicator strain. The following strains (162) were used as indicators: the principal indicator *Enterococcus avium* EA5 (from piglet feces), 8 vancomycin-resistant enterococci originating from food (kindly provided by Dr. Bírošová, Slovak Technical University in Bratislava, Faculty of Chemical and Food Technology), 4 staphylococci from raw goat milk (our strains), 11 staphylococci from different sources, 16 *S. felis* fecal strains from cats, 13 *S. chromogenes* fecal strains from cows, 1 *S. haemolyticus* strain from a cow, 2 strains of *S. sciuri* from feces, 30 canine strains of *S. pseudintermedius*, 34 strains of methicillin-resistant *S. aureus* from pigs, 5 human-origin *S. aureus* Met^R^ and 1 from a cow, 11 strains of *E. faecalis* from poultry feces, 9 canine fecal *E. faecium* strains highly resistant to aminoglycosides, and 17 human *E. faecium* HLAR strains (these strains were provided by Dr. Troscianczyk from the University of Lublin, Poland).

### 2.7. Freeze-Drying Process (Encapsulation) of Lactococci for Their Application in Yogurts

To encapsulate lactococci, the freeze-drying method was used as the simplest form of encapsulation [13], as previously described by Lauková et al. [17]. Lactococci (rifampicin-labeled variants) were grown in 300 mL of M17 broth (pH 6.9) overnight at 37 °C to reach an absorbance (A_600_) of up to 1.0. Then, the appropriate volume of grown lactococcal culture was mixed with skim milk in small flasks in a ratio of 1:1 (Simandl Company, Dolní Marklovice, Czech Republic). Flasks were frozen (at −80 °C), and freeze-drying was performed using a Micro Modulyo 230 freeze dryer (Thermo-electron corporation, Asheville, NC, USA). The cell count in the powder was checked using a standard microbiological method, meaning dilutions in Ringer solution (Merck, Darmstadt, Germany). Dilutions were spread on M17 agar with rifampicin (Difco) to count lactococci after incubation at 37 °C for 24–48 h.

### 2.8. Survival and Stability of Postbiotic (Bacteriocin) Active, Encapsulated Lactococci in Goat Milk Yogurts

The fresh goat milk white yogurts (150 g) used in the experiment were bought from the commercial market network. These yogurts are produced by Slovak producers and/or farms in central Slovakia for the commercial market network. As indicated on the product label, the yogurts contain commercial yogurt culture and 3.5% fat and possess the following parameters: energy 254 kJ/61 kcaL; fat 3.7 g, of which 2.2 g was saturated fatty acids; carbohydrates 3.9 g; sugar value 2.0 g. The protein content in the yogurts amounted to 3.7 g, with 0.26 g salt per 100 g of the product. Before application to the yogurts, the encapsulated lactococci were checked for cell counts using the standard microbiological dilution method. An amount of 0.5 g of each encapsulated *L. lactis* strain was applied to the experimental yogurts. The control yogurts were free of strains MK 2/2, MK2/7, and MK2/8. Before the application of lactococci, yogurt samples were diluted in peptone water, and the absence of non-required bacteria was confirmed by spreading dilutions on MacConkey agar (Difco-Becton Dickinson, Sparks, MD, USA) to check for coliforms. The yogurts were coliform-free. The yogurts were also checked for streptococci and lactic acid bacteria counts, which were expressed as colony-forming units per gram (log 10); they reached 5.1 and 8.1 CFU/g log 10, respectively. The cells of the applied lactococci were checked on M17 agar enriched with rifampicin (100 µg/mL) to differentiate them from other lactococci. The count of LAB was determined on MRS agar (Merck, Darmstadt, Germany). Sampling was performed 24 h after application and then on days 7, 10, and 14, when the microbiota evaluation was stopped because of the declared expiration time for goat milk yogurts. For plating, yogurts were sampled (1 g) and mixed in peptone water using a Stomacher-Masticator (IUL, Barcelona, Spain) in a ratio of 1:9. The appropriate dilutions were spread on the previously indicated media according to ISO. In addition, pH values were measured using a Checker-pH tester (Hanna Instruments Incorporation, Woonsocket, RI, USA). The initial pH ranged from 3.0 to 3.90. The yogurts were placed in the fridge during the test (14 days) period.

## 3. Results

### 3.1. Taxonomy and Enzyme Profile of Lactococci

Based on Blastn analysis, all three strains were taxonomically assigned to the species *Lactococcus lactis*, with a sequence identity percentage of 99.82% (Blastn 16S rRNA) for the strain MK2/2 compared with the sequence of the *Lactococcus lactis* strain in GenBank (MT545096). The accession number (AN) for strain MK2/2 is PQ158270. A sequence identity of 99.47% was determined for strain MK2/7 (AN PQ158271) compared with the sequence KX880980.1. Finally, strain MK2/8 was assessed with a sequence identity percentage of 99.82% compared to the sequence KX880977.1 (AN PQ158272).

Regarding the enzyme evaluation, lactococci did not produce damaging or beneficial enzymes, with only *L. lactis* MK2/7 producing 5 nmoL of the beneficial enzyme β-galactosidase. The tested lactococci did not produce the enzyme β-glucuronidase or N-acetyl-β-glucosaminidase. No production of trypsin and α-chymotrypsin greater than 5 nmoL was detected in the strains MK2/7 and MK2/8. The other enzymes were not produced.

### 3.2. Susceptibility to Antibiotics (Antimicrobials) and Biofilm-Forming Ability of Lactococci

The antibiotic phenotype of lactococci was detected by using the disk diffusion method. The strains were susceptible to clindamycin (2 µg), with a zone size from 17 to 23 mm. Regarding novobiocin (5 µg), the zone size ranged from 15 to 17 mm. In the case of penicillin (10IU), the susceptibility range was high (from 10 to 32 mm). Susceptibility to ampicillin (10 µg) was indicated in the strains, with zone sizes from 15 to 32 mm. Lactococci were susceptible to erythromycin (15 µg), with zone diameters of 20–26 mm; to streptomycin (25 µg), with inhibitory zones ranging from 11 to 15 mm; to rifampicin (30 µg), with zones sizes of 12–17 mm; and to vancomycin (30 µg), with zone sizes reaching 13–18 mm. Regarding kanamycin (30 µg), the zones measured 16–20 mm; for chloramphenicol (30 µg), zones were in the range of 23–27 mm; for ticarcillin (75 µg), the zones measured from 12 to 30 mm; and for gentamicin (120 µg), 17–23 mm. In general, the zone size ranges were balanced in lactococci; however, the largest inhibitory zone (the highest susceptibility) was measured in strain MK2/2 (12–32 mml). In the case of strain MK2/7, the zone sizes measured from 10 to 25 mm, and for strain MK2/8, the zone sizes measured from 11 to 27 mm.

Using the E-strip method, lactococci were susceptible to all antibiotics tested with different MICs (Table 1). Strain MK2/7 (in the case of Kan) and strain MK2/2 (regarding streptomycin) were neither susceptible nor resistant (Table 1).

Assessing biofilm-forming ability, strains MK2/2 and MK2/7 showed a low-grade ability to form biofilms (0.122 ± 0.005 for strain MK2/2; 0.120 ± 0.003 for strain MK2/7), and strain MK2/8 did not show biofilm-forming ability (0.099 ± 0.0). The biofilm-forming strain CCM 7316 formed biofilm with a value of 0.336 ± 0.219.

### 3.3. Postbiotic Activity Testing of Concentrated Substances Produced by Lactococci

The postbiotic potential of concentrated bacteriocin substances (CBs) produced by the tested lactococci is summarized in Table 2. As indicated previously, altogether, 162 indicator bacteria were included in testing: 46 enterococci and 116 staphylococci from various sources. The growth of 45 enterococcal strains out of 46 (97.8%) was inhibited by treatment with postbiotic substances from lactococci. The principal indicator strain, *E. avium* EA5, was inhibited using each CB (800 AU/mL). Enterococci inhibition was not dependent on their source of isolation (food-derived but also fecal strains) or on their species. Inhibition of the strains/species *E. faecalis*, *E. faecium*, *E. gallinarum*, and *E. casseliflavus* was detected, and inhibitory activity reached 400 AU/mL. The antimicrobial activity of postbiotic CBs from all three lactococci against enterococci was well balanced, except for the strain *E. faecalis* 220, which was not inhibited by MK2/2 CBs.

Among the staphylococcal indicators used (116), the growth of 94.8% was inhibited. In the case of MK2/2 CBs, 110 strains out of 116 staphylococci were inhibited. The same number of strains were inhibited using MK2/7 CBs (110 out of 116, Table 2), and 111 staphylococcal indicators were inhibited by MK2/8 CBs (95.7%); again, the postbiotic activity of all three CBs was well balanced, including one more inhibited strain in the case of MK2/8 CBs. The highest inhibitory activity reached 800 AU/mL. Regarding the inhibition of staphylococci species, the representatives of *S*. *pseudintermedius* were inhibited by all three CBs, with an inhibitory activity of up to 400 AU/mL (the highest activity was in the case of MK2/7 CBs). Almost all 16 strains of *S. felis* were inhibited; in the case of MK2/2 CBs, only the growth of two strains was not inhibited, and MK2/7 and MK2/8 CBs were only unable to inhibit one strain each: *S. felis* 62-1 and *S. felis* 16. The *S. chromogenes* strains were inhibited by the use of all three CBs; however, only low inhibitory activity (100 AU/mL) was measured. Surprisingly, *S. aureus* strains isolated from human samples (11) were also inhibited, with an inhibitory activity of up to 400 AU/mL. In the case of food-derived *S. aureus* strains (milk, cheese), inhibition was also noted, with activity of up to 200 AU/mL. Only one strain was not inhibited using MK2/7 CBs. When methicillin-resistant staphylococci from pig feces were treated with lactococcal postbiotic active CBs, at least 31 out of 34 strains were inhibited (Table 2) (100–400 AU/mL). *S. aureus* from cow feces was inhibited by three CBs (100 AU/mL), as were *S. sciuri* of human origin and *S. haemolyticus* from a cow (155SHLK39). And, finally, the *S. arlettae*, *S. delphini*, and *S. schleiferi* strains isolated from raw goat milk as postbiotic active lactococci were also inhibited. The most susceptible among these three species strains were the *S. arlettae* strains (up to 800 AU/mL).

### 3.4. Survival and Stability of Postbiotic Active, Encapsulated Lactococci in Goat Milk Yogurts

Before starting to supplement the yogurts, the microbial backgrounds in the yogurts were analyzed to determine the impact on amylolytic cocci and lactic acid bacteria (LAB). The pH of the yogurts was the same: 3.30 ± 0.1–3.90 ± 0.1. The initial value of the encapsulated MK2/2 strain reached 7.95 cfu/g (log 10) in yogurt. The count for strains MK2/7 and MK2/8 reached 7.84 cfu/g. Strain MK2/7 (4.94 ± 0.2 cfu/g) was the most established strain in the yogurts after 24 h, followed by strain MK2/2 (2.65 ± 0.1 cfu/g, log 10; Table 3). Strain MK2/8 was detected in an amount of 1.30 ± 0.1 log 10 cfu/g after 24 h. However, on day 7, strain MK2/8 had increased in the experimental yogurt, with a difference of 2.69 log cycles. Strain MK2/2 was increased with a difference of 1.95 log cycles, and strain MK2/7 was increased with a difference of 1.62 log cycles. On day 10, strain MK2/2 had increased to 5.85 ± 0.3 cfu/g (log 10) in the yogurts, with a difference of 1.25 log cycles compared to day 7. At the same time, strain MK2/7 increased in the yogurts with a difference of 0.78 log cycles, and strain MK2/8 increased with a difference of 2.1 log cycles. Finally, on day 14, the count of strain MK2/2 in yogurts was almost the same as on day 10; it reached 5.78 ± 0.6 cfu/g (log 10). The count of MK2/7 was not higher than on day 10, and it reached 7.0 cfu/g (log 10). However, the highest count was reached by strain MK2/8 (8.1 cfu/g, log 10). The counts of amylolytic cocci were correlated with the counts of the applied strains after 24 h; therefore, they were the lowest in yogurts enriched with strain MK2/8, followed by strain MK2/2, and the highest in yogurts with strain MK2/7 (Table 3). Their counts also increased in the control yogurts. On day 7, the highest increase in amylolytic cocci was noted in yogurts with strain MK2/8 (difference of 3.83 log cycles), while in yogurts with strain MK2/2, amylolytic cocci were almost at the same count. In yogurts with strain MK2/7, amylolytic cocci increased with a difference of 1.4 log cycles. On day 10, amylolytic cocci counts in the control yogurts were almost the same or slightly increased. Their counts were almost the same in the cases of strains MK2/2, MK2/7, and MK2/8. Finally, on day 14, amylolytic cocci reached 9.51 ± 0.6 cfu/g in the control yogurts; an increase was also shown in yogurts with strain MK2/2 (9.1 cfu/g) and in yogurts with strain MK2/7 (10.1 ± 1.2 cfu/g). The count of strain MK2/8 was almost at the same level as on day 10 (Table 3). The counts of lactic acid bacteria were high, and they continually increased over the experimental period (Table 3). The pH of the yogurts corresponded with the microbiota count and was not negatively influenced.

## 4. Discussion

The species *Lactococcus lactis* is a bacterium extensively used in the dairy industry and possesses different functions in dairy fermentation, such as the production of lactic acid from lactose, citric acid fermentation, and the hydrolysis of casein [1]. Moreover, representatives of this species that produce bacteriocin substances can exert an inhibitory effect against several spoilage bacteria [29]. For example, Sanca et al. [30] presented the probiotic strain *L. lactis* subsp. *lactis* L2 with inhibitory activity against pig pathogens in vitro. Lacticin 3147, a two-component, broad-host-range bacteriocin produced by *L. lactis* subsp. *lactis* DPC3147, isolated from Irish kefir-like grains, was found to act on the cytoplasmic membrane of sensitive cells, forming pores. It inhibits listeriae, staphylococci, streptococci, and clostridia [31] but not Gram-negative bacteria. The anti-staphylococcal effect of lactococcal CBs was also demonstrated in vitro in this study, involving various staphylococcal species strains. In addition, enterococci were also inhibited. This means that lactococcal CBs showed an inhibitory effect against Gram-positive bacteria; however, up to now, the inhibitory effect against Gram-negative indicator strains has not been tested. Even et al. [32] reported that the application of *L. lactis* in the dairy industry can prevent *S. aureus* food poisoning by controlling enterotoxin production. Wu et al. [2] reported that *L. lactis* strains RWP-3 and RWP-7 prevented bacterial infections and optimized the intestinal microbiota of humans and animals as well through their ability to inhibit the growth of pathogens. For example, milk fermented by *L. lactis* NRRL B-50571 and B-50572 is able to reduce blood pressure and blood lipids [33]. The lactococci presented in this study showed sufficient survival and stability in goat milk yogurts when applied in encapsulated (freeze-dried) form. Akbar and Anal [29] isolated an *L. lactis* subsp. *lactis* strain from fermented milk, which was used as a bio-control agent against *S. aureus*.

The strains for fortification should fulfill safety requirements. The enzyme profile of a beneficial strain is one of the most important parameters and also one of the markers of the strain’s characteristics and safety. Based on the enzyme type, its production and/or lack of production can indicate whether it is beneficial. The lactococci tested did not produce damaging enzymes, which can indicate their safe habitat. Some damaging enzymes, e.g., N-acetyl-β-glucosaminidase and α-chymotrypsin, serve as disease markers. Bacterial β-glucuronidase can even play a role in colon cancer. Therefore, the zero value of that enzyme measured in the tested lactococci also contributes to their safe habitat. Similarly, the beneficial and postbiotic (bacteriocin) active strain *E. durans* ED26E/7 isolated from ewe milk lump cheese did not produce that enzyme [34].

To distinguish strains susceptible to antibiotics and/or those with intrinsic or acquired resistance, antibiotic profile testing is required, including MIC evaluation. Postbiotic active lactococci were found to be susceptible to antibiotics. Similarly, Floréz et al. [35] tested strains of *L. lactis* isolated from dairy, and they found them to be susceptible to erythromycin, chloramphenicol, vancomycin, and other tested antibiotics. Lactococci are easily adaptable bacteria to specific environmental determinants, which can make them suitable for several uses [2]. The important factors in the case of each applied strain are the form used as well as its survival and stability in the product.

*L. lactis* can be used, e.g., as a natural preservative and anti-botulism agent in cheeses [36]. Yogurt is a popular fermented dairy product, and that produced from goat milk especially possesses nutritional and nutraceutical benefits. For example, the protein present in yogurt can be easily digested. It is proven to reduce cholesterol levels. It can lessen the risk of type 2 diabetes by improving insulin sensitivity and glucose tolerance. It can also improve bone density. These are all points that have led to the use of yogurts as functional foods, especially those enriched with beneficial microbiota. In our previous studies, selected beneficial strains, such as *Lactiplantibacillus plantarum* LP17L/1 and/or *Lacticaseibacillus paracasei* LPa12/1 [17,21], were checked for their additional benefits in model Balb/c mice and/or food animals, such as broiler rabbits, based on their influence on health status and/or, e.g., immunological parameters. No mortality was detected in mice. In the case of broiler rabbits and the LP17L/1 strain, the total lactic acid bacteria and amylolytic streptococci were significantly increased (*p* < 0.001). Lower GPx values were measured in the experimental rabbits in comparison with control animals, which means that this strain did not induce oxidative stress. Phagocytic activity in the blood of the rabbits was not negatively influenced [17,21]. Moreover, in the case of the LP17L/1 strain, the detected protective effect against *Trichinella spiralis* infection was associated with the increased oxidative metabolism of peritoneal macrophages, which activated the metabolic activity of macrophages during the migration of newborn larvae [37]. In each of these studies, the application form and stability of the strains are of paramount interest. The encapsulation of beneficial strains is a useful application form that fulfills requirements for the stability of strains in applied environments. Sufficient growth of the LPa12/1 strain in goat milk yogurts was reported in our previous study [21]. In the future, the lactococci studied in this study will also be checked to evaluate their functional character.

In spite of the fact that some strains of *L. lactis* are well established as commercial probiotics, there is still a need to refine their benefits. Among them is their postbiotic activity. Rajneesh Thakur et al. [38] reported that milk is considered a significant source of bioactive peptides (postbiotics) and has the potential to be utilized in the production of nutritional supplements owing to its beneficial health impacts on humans. Anticarcinogenic properties were also mentioned. Therefore, our study is a promising contribution showing the benefits of goat milk and its products fortified with beneficial postbiotic lactococci.

## 5. Conclusions

The concentrated antimicrobial substances of three postbiotic (bacteriocin) active strains of the species *Lactococcus lactis* (isolated from Slovak raw goat milk) inhibited the growth of 97.8% enterococcal indicators with an inhibitory activity of up to 800 AU/mL. Among 116 staphylococcal indicators, the growth of 94.8% was inhibited. The inhibitory activity was not influenced by the species strain. The taxonomy of lactococci was established using Blastn 16S rRNA analysis, reaching sequence identity percentages in the range from 99.47% to 99.82% compared with the sequences of the reference *Lactococcus lactis* strains. Lactococci did not produce damaging enzymes. *L. lactis* MK2/7 produces 5 nmoL of the beneficial enzyme β-galactosidase. Strains MK2/2 and MK2/7 showed low-grade positive biofilm-forming ability. Lactococci were mostly susceptible to the tested antibiotics. The fortification of yogurts made from goat milk with encapsulated lactococci showed their sufficient stability and survival, with the highest adaptation observed for the MK2/8 strain. The results obtained indicate the high potential of postbiotic active lactococci for their practical use as functional additives. Further clinical studies are required to test the final effects on consumers.

## Figures and Tables

**Table 1:** Summary of susceptibility to antibiotics in lactococci using the E-test (MIC, minimum inhibitory concentration, is expressed in µg).

Strain	Kan	Str	Pnc	Chc	Ery	Rif	Tc	Va	Gn	Amp
MK2/2	4/S	96/S/R	0.094/S	5/S	0.047/S	6/S	0.25/S	0.38/S	2/S	0.016/S
MK2/7	96/S/R	24/S	0.38/S	6/S	0.064/S	0.08/S	0.25/S	1.5/S	8/S	0.19S
MK2/8	10/S	3/S	0.064/S	12/S	0.50/S	0.08/S	0.38/S	3/S	0.094/S	0.094/S

Kan: kanamycin; Str: streptomycin (0.064–1024 µg/mL); Pnc: penicillin (0.016–256 µg/mL); Chc: chloramphenicol (0.016–256 µg/mL); Ery: erythromycin (0.015–256 µg/mL); Rif: rifampicin (0.032–32 µg/mL); Tc: tetracycline (0.016–256 µg/mL); Va: vancomycin (0.016–256 µg/mL); Gn: gentamicin (0.064–1024 µg/mL); Amp: ampicillin (0.016–256 µg/mL); S: susceptible; S/R: dubious. Explanation: 4/S means MIC = 4 µg, and it is susceptible.

**Table 2:** Inhibitory activity of postbiotic substances from lactococci (expressed in arbitrary units per milliliter, AU/mL).

Indicators	MK2/2	MK2/7	MK2/8
*E. avium* EA5	1/1, 800	800	800
*E. gall*.	3/3, 100, 400, 100	100, 400, 100	100, 200, 100
*E. cassel*.	4/4, 100, 100, 100, 100	100, 100, 100, 100	100, 0, 100, 100
*E. faecium* VRE13	1/1, 100	100	100
*E. faecalis*	11/10, 100–200	11/11, 100–400	11/11, 100–200
*E. faecium*	9/9, 100–400	9/9, 100–400	9/9, 200–400
*E. faecium*	17/17, 100–200	17/17, 200–400	17/17, 200–400
*S. felis*	16/14, 100–400	16/15, 100–800	16/15, 100–800
*S. chromogenes*	13/13, 100	13/13, 100	13/13, 100
*S. aureus*, *human*	6/5, 100	6/4, 100–200	6/6, 100
*S. aureus*, *milk*, *cheese*	4/4, 100	4/3, 100–200	4/4, 100–200
*S. aureus*, *human*	5/5, 100–200	5/5, 100–400	5/5, 100–200
*S. aureus*, *cow*	1/1, 100	1/1, 100	1/1, 100
*S. aureus*, *pig*	34/32, 100	34/33, 100–400	34/31, 100–200
*S. arlettae*	2/2, 400, 800	2/2, 400, 800	2/2, 400
*S. schleiferi*	1/1, 400	1/1, 100	1/1, 200
*S. delphini*	1/1, 400	1/1, 800	1/1, 800
*S. sciuri* human	2/1, 100	2/1, 100	2/1, 100
*S. haemolyticus* cow	1/1, 100	1/1, 100	1/1, 100
*S. pseudintermedius*	30/30, 100–200	30/30, 100–400	30/30, 100–200

*E. gall*.: *E*. *gallinarum* VRE10, *E. gallinarum* VRE18, *E. gallinarum* VRE16; *E. faecium* VRE13: food-derived strain; *E. faecalis*: isolated from canine feces; *E. faecium* HLAR 9/9: from poultry feces; *E. faecium*: human multiresistant strains; *S. felis*: isolated from feces of cats; *S. chromogenes:* feces of cows; *S. aureus*: sources are human, milk, and cheese; *S. arlettae*, *S. schleiferi*, and *S. delphini*: from raw goat milk; *S. sciuri*: from mastitis; 34 MRSA *S. aureus* from pig feces, 5 human *S. aureus* strains, 1 *S. aureus* from cow; x/x means number of tested strains/number of inhibited strains; e.g., 800 means inhibitory activity in AU/mL. Some strains were kindly provided by our colleagues as indicated in Materials and Methods.

**Table 3:** Goat milk yogurts with fortification of postbiotic active lactococci, their stability, and their survival in yogurts (in colony-forming unit per gram, cfu/g log 10 ± SD).

Sampling	pH	Lactococci Tested	Amylolytic Cocci	LAB
Control/24	3.35 ± 0.1	nt	7.06 ± 0.1	7.1 ± 0.0
MK2/2	3.30 ± 0.0	2.65 ± 0.1	7.1 ± 0.0	8.96 ± 0.5
MK2/7	2.99 ± 0.1	4.94 ± 0.2	8.17 ± 0.0	8.1 ± 0.0
MK2/8	3.34 ± 0.1	1.30 ± 0.1	5.1 71 ± 0.0	6.95 ± 0.7
Control/7 day	3.33 ± 0.1	nt	8.38 ± 0.5	8.46 ± 0.5
MK2/2	3.21 ± 0.1	4.60 ± 1.1	7.83 ± 1.0	8.61 ± 065
MK2/7	2.93 ± 0.1	6.56 ± 0.5	10.1 ± 1.5	8.30 ± 0.3
MK2/8	3.35 ± 0.1	4.0 ± 0.0	8.93 ± 085	7.48 ± 0.1
Control/10 day	3.33 ± 0.1	nt	8.98 ± 0.7	9.18 ± 0.5
MK2/2	3.21 ± 0.1	5.85 ± 0.3	7.1 ± 0.0	9.52 ± 0.1
MK2/7	2.92 ± 0.1	7.34 ± 0.5	10.5 ± 1.5	8.91 ± 0.2
MK2/8	3.35 ± 0.1	6.1 ± 0.0	8.1 ± 0.0	9.48 045
Control/14 day	3.33 ± 0.1	nt	9.51 ± 0.6	8.70 ± 0.5
MK2/2	3.21 ± 0.1	5.78 ± 0.6	9.1 ± 0.0	9.20 ± 1.0
MK2/7	3.29 ± 0.1	6.93 ± 0.8	10.1 ± 1.2	9.69 ± 0.9
MK2/8	3.35 ± 0.1	8.1 ± 0.0	8.88 ± 0.9	9.41 ± 0.8

nt—not tested; LAB—lactic acid bacteria.

## Data Availability

The complete data produced in this study are available to research applicants.
